# Inhibition of phosphorylated calcium/calmodulin-dependent protein kinase IIα relieves streptozotocin-induced diabetic neuropathic pain through regulation of P2X3 receptor in dorsal root ganglia

**DOI:** 10.1007/s11302-021-09829-z

**Published:** 2022-01-01

**Authors:** Xiao-fen He, Yu-rong Kang, Xue-yu Fei, Lu-hang Chen, Xiang Li, Yi-qi Ma, Qun-qi Hu, Si-ying Qu, Han-zhi Wang, Xiao-mei Shao, Bo-yi Liu, Yi-Liang, Jun-Ying Du, Jian-qiao Fang, Yong-liang Jiang

**Affiliations:** 1 Key Laboratory of Acupuncture and Neurology of Zhejiang Province, Department of Neurobiology and Acupuncture Research, The Third Clinical Medical College, Zhejiang Chinese Medical University, Hangzhou, Zhejiang 310053 People’s Republic of China; 2 Zhejiang Chinese Medical University, Hangzhou, Zhejiang 310053 People’s Republic of China; 3 Department of Acupucture, the Rehabilitation Hospital Affiliated To Tongxiang Health School, Jiaxing, Zhejiang 314500 People’s Republic of China

**Keywords:** Diabetic neuropathic pain, p-CaMKIIα, Streptozotocin, DRG, KN93, P2X3 receptor

## Abstract

Diabetic neuropathic pain (DNP) is frequent among patients with diabetes. We previously showed that P2X3 upregulation in dorsal root ganglia (DRG) plays a role in streptozotocin (STZ)-induced DNP but the underlying mechanism is unclear. Here, a rat model of DNP was established by a single injection of STZ (65 mg/kg). Fasting blood glucose was significantly elevated from the 1^st^ to 3^rd^ week. Paw withdrawal thresholds (PWTs) and paw withdrawal latencies (PWLs) in diabetic rats significantly reduced from the 2^nd^ to 3^rd^ week. Western blot analysis revealed that elevated p-CaMKIIα levels in the DRG of DNP rats were accompanied by pain-associated behaviors while CaMKIIα levels were unchanged. Immunofluorescence revealed significant increase in the proportion of p-CaMKIIα immune positive DRG neurons (stained with NeuN) in the 2^nd^ and 3^rd^ week and p-CaMKIIα was co-expressed with P2X3 in DNP rats. KN93, a CaMKII antagonist, significantly reduce mechanical hyperalgesia and thermal hyperalgesia and these effects varied dose-dependently, and suppressed p-CaMKIIα and P2X3 upregulation in the DRGs of DNP rats. These results revealed that the p-CaMKIIα upregulation in DRG is involved in DNP, which possibly mediated P2X3 upregulation, indicating CaMKIIα may be an effective pharmacological target for DNP management.

## Introduction

Diabetes patients often develop diabetic neuropathic pain (DNP) [1, 2]. DNP symptoms include paresthesia, hyperalgesia, allodynia, and spontaneous pain [3, 4]. A painful sensation is transmitted by dorsal root ganglia (DRG) from peripheral afferents to the central nervous system [5]. Sensitization of DRG neurons and associated nerve fibers is suggested as a major cause of DNP [6, 7]. DNP markedly reduces patients’ quality of life, which may cause withdrawal from social events and depression [8–10]. Since DNP pathogenesis is not well understood, DNP treatment is a challenging.

Streptozotocin (STZ), a glucosamine-nitrosourea compound obtained from Streptomyces achromogenes, has been applied in research to establish animal models diabetes to explore diabetes and its complications, including DNP. STZ-induced hyperglycemia is reported to contribute to hyperalgesia development [11, 12].

P2X receptors are abundant in DRG neurons [13, 14]. P2X3 sensitization is reported to cause inflammatory pain and neuropathic pain [11, 15]. Previously, we have shown that P2X3 upregulation in DRG influences STZ-induced DNP [12, 16], but the mechanisms underlying P2X3 upregulation in DRG during DNP are unclear.

Calcium/calmodulin-dependent protein kinase II (CaMKII) is encoded by one of four genes (α, β, γ, and δ)[17]. The γ and δ isoforms are ubiquitously expressed whereas α and β isoforms are expressed abundantly in nerve cells [18]. CaMKIIα is the most abundant isoform of neuronal CaMKII [19]. CaMKII participates in processing of nociceptive signals in primary sensory neurons of the DRG [20, 21]. CaMKII is activated by calcium/calmodulin binding, which frees the catalytic domain to auto-phosphorylate the kinase domain on Thr286 or Thr287 [22, 23]. Phosphorylated CaMKIIα levels are elevated in type 1 diabetic animals and are accompanied by pain-related behaviors [24]. CaMKII is reported to regulate purinergic signaling via an intracellular pathway, that modulates the efficiency and stability of P2X3 [25]. Suggesting that p-CaMKIIα may mediate P2X3 upregulation in DRG undering DNP.

Here, we used a STZ-induced rat model of DNP to assess mechanical allodynia, thermal hyperalgesia, and p-CaMKIIα expression in DRG. We find that p-CaMKIIα and P2X3 are co-expressed in DRG during DNP. The effects of KN93, a CaMKII inhibitor, on pain-related behavior and p-CaMKIIα and P2X3 receptor expression in DRG were also studied.

## Materials and methods

### Animals

Adult male Sprague–Dawley rats (180–220 g) were purchased from Shanghai Laboratory Animal Center of Chinese Academy of Sciences (SCXK (hu) 2018–0006). All animals were housed in a temperature-controlled environment at 25 ± 2 °C with 12 h light/dark cycles and 55% ± 5% humidity with ad libitum access to food and water. The study was approved by the Animal Welfare Committee of Zhejiang Chinese Medical University (IACUC – 20180723–08).

### Induction of type 1 diabetic neuropathic pain

To induce diabetes, the rats were fasted for 16 h and then administered with a single dose of STZ (Sigma, USA) at 65 mg/kg body weight in 0.1 mol/L sodium citrate (pH = 4.5) [26, 27]. Rats with fasting blood glucose (FBG) > 13.9 mmol/L [28, 29] and exhibiting pain behavior two weeks after injection were considered as successful DNP models.

### Experimental design and animal grouping

**Fig. 1:**
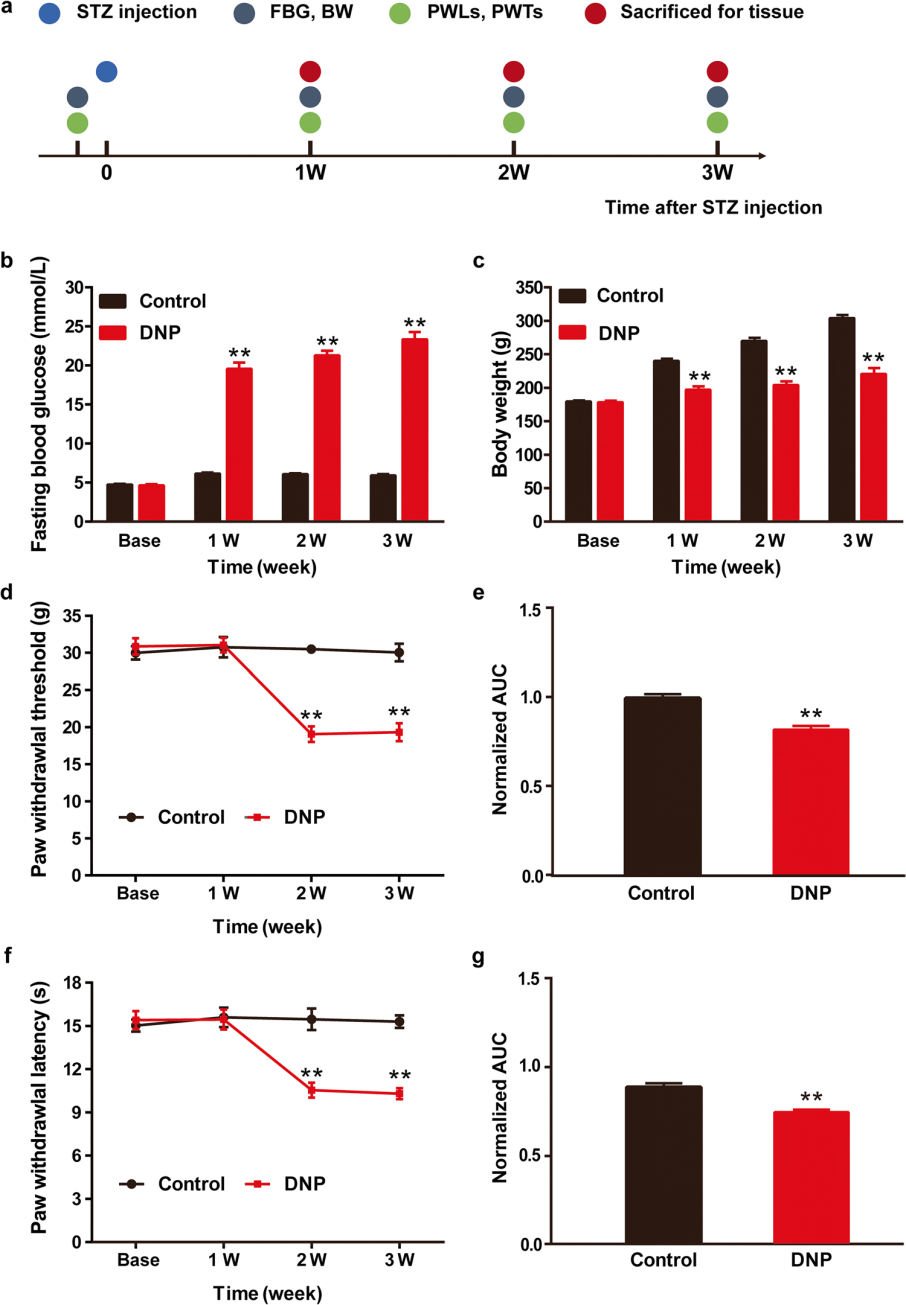
STZ induces diabetic neuropathic pain in rats. (**a**) Schematic of protocol for establishing the DNP model. (**b**) Effects of STZ on FBG at various time-points. (**c**) Effects of STZ on BW at various time-points. (**d**) Effects of STZ on PWT at various time-points. (**e**) Normalized AUC analysis of (**d**). (**f**) Effects of STZ on PWL at various time-points. (**g**) Normalized area under the curve (AUC) analysis of (**f**). ^**^P < 0.01 vs. Control group. *n* = 6

Our study was divided into 2 parts. In Part 1, we assessed the development of DNP after STZ injection using behavioral assays and evaluated the role of CaMKIIα and p-CaMKIIα in L4-L6 DRGs of DNP rats. Experimental rats were randomly allocated to Control group (12 rats) sacrificed 3 weeks after sodium citrate buffer injection for tissues, and DNP group (36 rats). Of the DNP rats, 9 were sacrificed 1 week after STZ injection, 9 were sacrificed 2 weeks after STZ injection, 12 were sacrificed 3 weeks after STZ injection and tissues harvested, and 6 rats died or failed to successfully model DNP. Paw withdrawal thresholds (PWT) and paw withdrawal latency (PWL) were recorded according to the schedule (Fig. 1a).

In Part 2, we examined the involvement of p-CaMKIIα in DNP through treating with KN93, a CaMKII inhibitor. To this end, rats were randomly divided into 4 groups: (1) Control + vehicle group, (2) DNP + vehicle group, (3) DNP + 25 nmol KN93 group, (4) DNP + 50 nmol KN93 group. Pain behavioral tests were then performed according to the schedule (Fig. 7a). Out of 36 rats injected with STZ, 30 successfully modeled DNP while 3 died and 3 were unsuccessfully modeled.

### Fasting blood glucose and body weight measurements

Fasting blood glucose (FBG) and body weight (BW) were recorded at the beginning of the study and monitored weekly. Rats were fasted for 8 h, blood samples drawn from the tail vein, and FBG measured using a glucometer (Roche, Germany).

### Measurement of paw withdrawal threshold

The (PWT) was determined before STZ injection and 1, 2, and 3 weeks after STZ injection. The PWT was determined using a dynamic plantar aesthesiometer (37450, Ugo Basile, Italy) as detailed previously [16]. Briefly, rats were placed in Plexiglas cubicles on a wire mesh platform for a 15-min adaptation. A stimulating probe was then positioned under their left hind paw and an increasing vertical force (continuous increase from 0 to 50 g in 20 s) applied. The instrument recorded the force that triggers limb withdrawal and the tolerance threshold was given by the mean of 3 readings. Experimenters were blinded to the experimental design.

### Measurement of the paw withdrawal latency

The paw withdrawal latency (PWL) was determined before STZ injection and 1, 2, and 3 weeks after STZ injection, respectively. PWL was determined using noxious thermal stimulation (37370, Ugo Basile, Italy) as described before [30]. After adaptation, radiant heat was applied under the left hind paw and the time to paw withdraw recorded immediately to the nearest 0.1 s. Tolerance latency was given by the 3 measurements. The cut-off time for heat stimulation was 20 s. Hind paws were tested at 5-min intervals. Experimenters were blinded to the experimental design.

### Western blot analysis

L4-L6 DRGs were harvested, lysed in RIPA buffer and cleared by centrifugation at 12,000 rpm for 20 min. After quantification, the protein samples (25 ug) were resolved by 8% SDS-PAGE before transfer onto PVDF membranes. Membranes were blocked with 5% milk-TBST for 1 h at 37 ℃ and then incubated with rabbit anti-p-CaMKIIα (ab5683, 1:1000, Abcam, USA), mouse anti-CaMKIIα (50049, 1:1000, CST, USA), and mouse anti-β-actin (12262,1:5000, CST, USA) at 4 °C overnight. Membranes were washed with TBST for 5–10 min and then incubated with HRP-conjugated anti-rabbit (7074, 1:5000, CST, USA) or anti-mouse (7076,1:5000, CST, USA) IgG for 2 h at 37 °C. Signal was then developed by enhanced chemiluminescence (Beyotime, Shanghai, China) following manufacturer instructions. The protein bands were analyzed using ImageJ software and normalized to β-actin.

### Immunofluorescence

Rats were anaesthetized by intraperitoneal injection of sodium pentobarbital (80 mg/kg) and perfused with saline followed by 4% paraformaldehyde respectively. L4-L6 DRGs were then quickly collected from sacrificed rats and fixed in 4% formaldehyde for 4 h. The collected tissues were then cryoprotected overnight in 15% and 30% sucrose solution at 4 °C until they had sunk to the bottom. They were then frozen embedded in optimum cutter temperature (OCT)-compound (SAKURA, Torrance, CA, USA), serially sectioned at 10 μm using a cryomicrotome (NX50 HOP, Thermo, Germany) and mounted onto gelatin-coated glass slides.

To assess p-CaMKII α expression in L4-L6 DRG neurons, sections were permeabilized with 0.1% TBST and blocked with 10% normal donkey serum for 1 h before incubation with rabbit anti-p-CaMKIIα (ab5683, 1:800, Abcam, USA) and mouse anti-NeuN (ab104224, 1:500, Abcam, USA) at 4 °C, overnight. They were then incubated with Alexa Fluor 488 donkey anti-rabbit IgG (711–545-152, 1:800, Jackson, USA) and Alexa Fluor 647 donkey anti-mouse IgG (715–605-150, 1:800, Jackson, USA) at 37 °C for 1 h and imaged on a fluorescence microscope (Zeiss Imager M2, Germany). Fluorescence intensity was then analyzed on ImageJ software (3 to 5 images were measured for each DRG).

To assess co-expression between P2X3 and p-CaMKII α in L4-L6 DRGs, sections were permeabilized with 0.1% TBST and blocked with 10% normal goat serum for 1 h. They were then incubated with anti-p-CaMKIIα (phospho T286, ab5683, 1:800, Abcam, USA) and guinea pig anti-P2X3 (GTX10267, 1:500, GeneTex, USA) at 4 °C, overnight. Next, they were incubated with Alexa Fluor 488 goat anti-rabbit IgG (ab150077, 1:800, abcam, USA) and Alexa Fluor 647 goat anti-guinea pig IgG (ab150187, 1:800, Abcam, USA) secondary antibodies at 37 °C for 1 h and imaged on a fluorescence microscope.

### Drug administration

KN93 (422708-5MG, Sigma-Aldrich, USA), a specific CaMKII inhibitor, was dissolved in sterile 0.9% saline and diluted to specific concentrations immediately before each experiment. Rats were then injected with KN93 (25 nmol or 50 nmol) in the ventral surface of each hind paw. The control + vehicle and DNP + vehicle groups were injected with the same volumes of 0.9% saline.

### Statistical analysis

Data were analyzed using SPSS version 21.0 and were expressed as mean ± SEM. Independent-sample *t*-test was used to compare 2 groups. One-way ANOVA with least significant difference (LSD) as post hoc test was used to compare > 2 groups. For the behavioral tests, two-way repeated-measures ANOVA, followed by Bonferroni’s post hoc test, was used. *P* < 0.05 was considered statistically significant.

## Result

### STZ induces diabetic neuropathic pain in rats

In STZ-induced diabetic rats, FBG levels were markedly elevated in the 1st week of STZ (65 mg/kg) injection and persisted until the 3^rd^ week when compared to control rats (*p* < 0.01) whose FBG remained at baseline levels (Fig. 1b). Relative to control rats whose body weight continued to rise, growth rate was significantly slowed in DNP rats (*p* =  < 0.01, Fig. 1c). Relative to the control group, DNP rats had significantly lower PWTs and PWLs in the 2nd week and this persisted until the 3rd week (*P* < 0.01, respectively, Fig. 1d–g). These results indicated that DNP model was successfully established on the 2nd week after STZ injection.

### CaMKII α and p-CaMKII α protein levels in L4-L6 DRGs after STZ injection

**Fig. 2:**
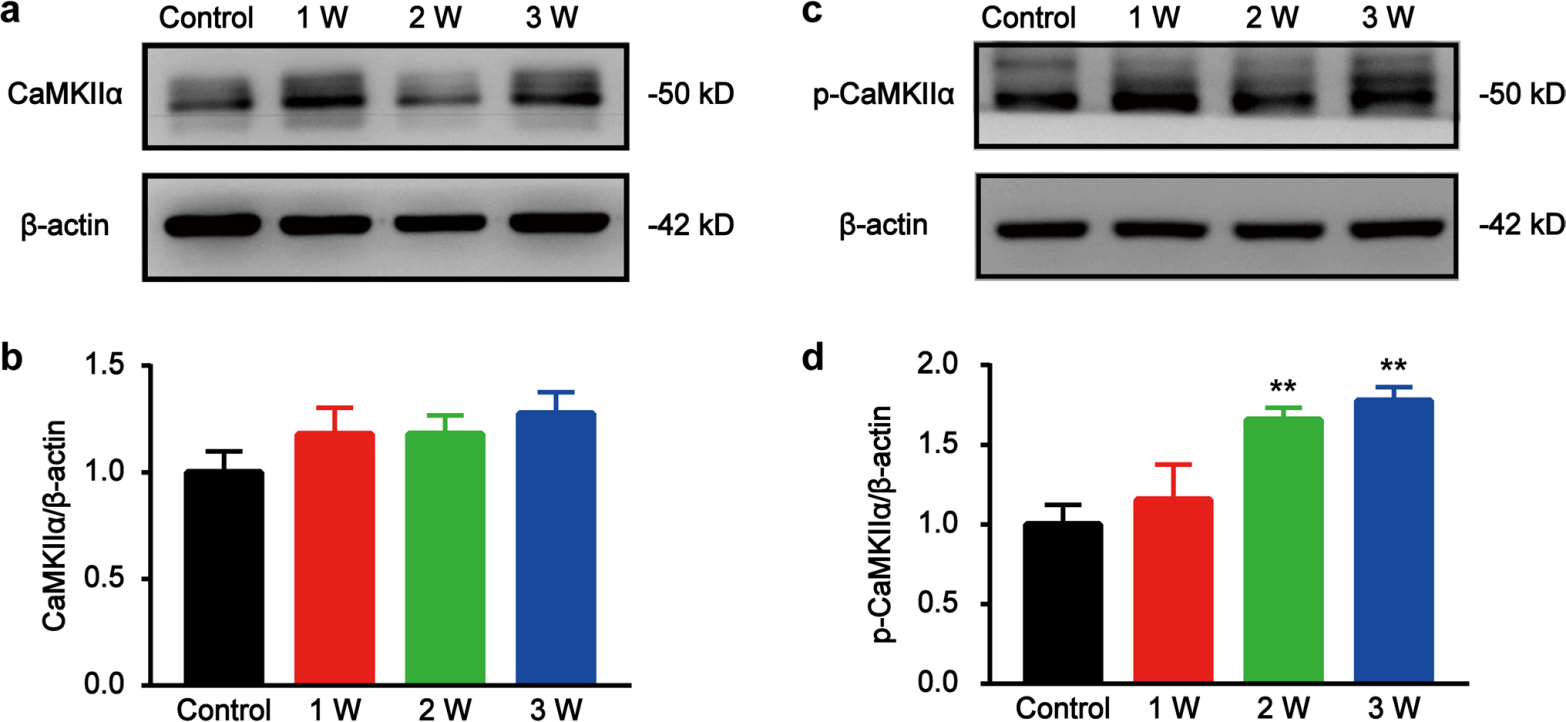
Western blot analysis of CaMK II α and p-CaMK IIα levels in DRGs of STZ-induced diabetic rats. (**a**) Representative western blot image of CaMK IIα levels. (**b**) Relative protein levels of CaMK IIα in rat L4-6 DRGs. Data are presented as mean ± SEM, *n* = 6. (**c**) Representative western blot images of p-CaMK IIα levels. (**d**) Relative p-CaMK IIα protein levels in rat L4-6 DRGs. ^∗∗^*P* < 0.01, vs. Control group. Data are presented as mean ± SEM. *n* = 6

Western blot analysis of the CaMKIIα and p-CaMKIIα levels in L4-L6 DRGs revealed that p-CaMKII α levels significantly increased in STZ-induced diabetic rats (Fig. 2c, d), while CaMKII α level did not change significantly (Fig. 2a, b). Indicating that p-CaMKIIα was upregulated in DRG, which is consistent with DNP model establishment upon STZ injection.

### Co-expression of p-CaMKII α/NeuN in L4-L6 DRGs

**Fig. 3:**
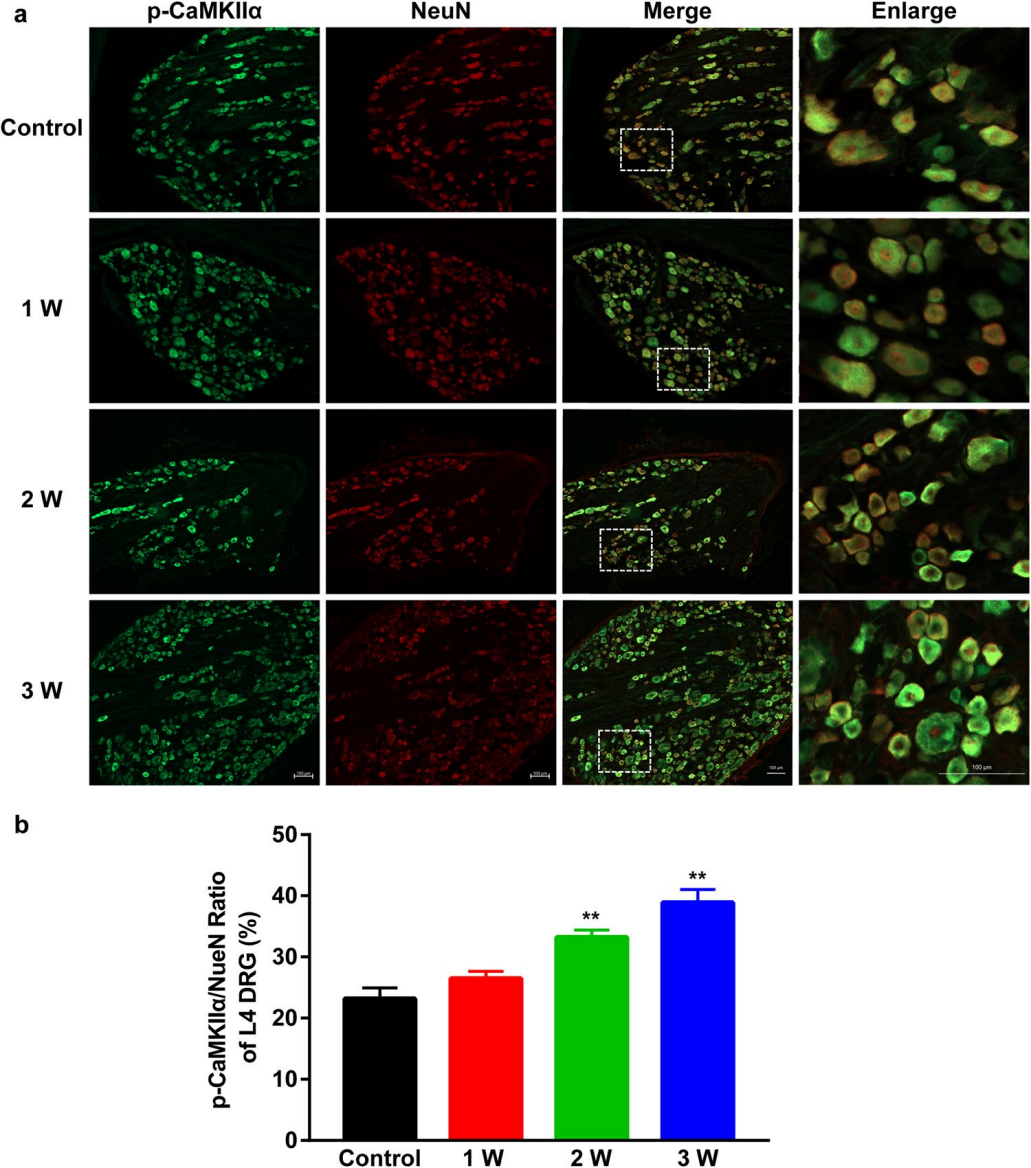
(**a**) Representative IF images of p-CaMK II α (green) expression in neurons (red, NeuN) in L4 DRG of DNP rats. Scale bar 100 µm. (**b**) p-CaMK II α/NeuN ratio in L4 DRGs in Control, 1 W, 2 W, and 3 W group. ^**^P < 0.01 vs. Control group. Data are presented as mean ± SEM. *n* = 3

**Fig. 4:**
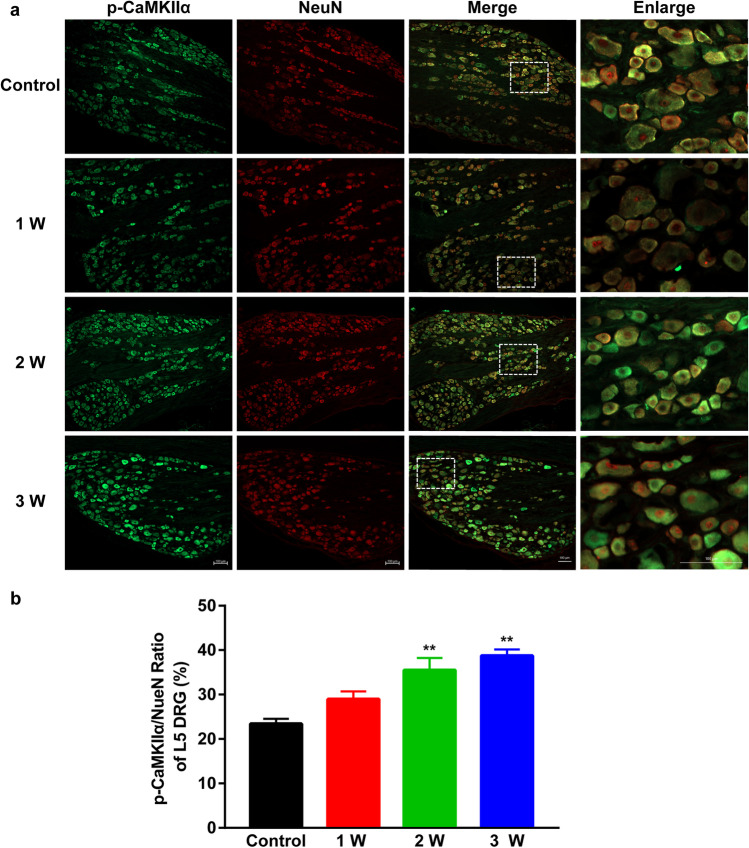
(**a**) Representative IF images of p-CaMK IIα (green) expression in neurons (red) in L5 DRG of DNP rats. Scale bar 100 µm. (**b**) p-CaMK IIα/NeuN ratio in L5 DRGs in Control, 1 W, 2 W, and 3 W group. ^**^P < 0.01 vs. Control group. Data are presented as mean ± SEM. *n* = 3

**Fig. 5:**
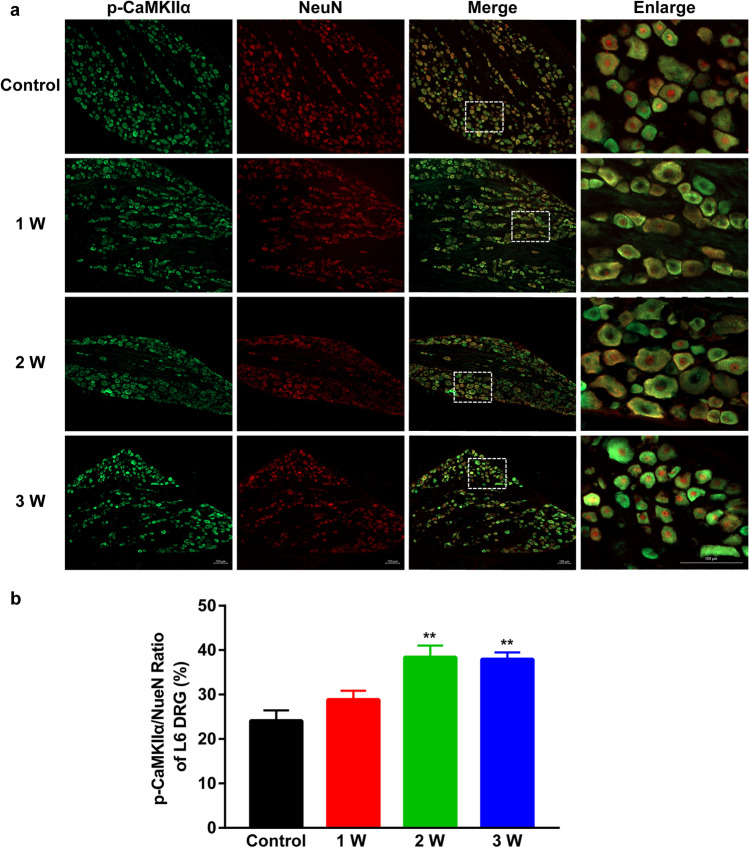
(**a**) Representative IF images of p-CaMK IIα positive (green) expression in neurons (red) in L6 DRG of DNP rats. Scale bar 100 µm. (**b**) p-CaMK IIα/NeuN ratio in L6 DRGs in Control, 1 W, 2 W, and 3 W group. ^**^P < 0.01 vs. Control group. Data are presented as mean ± SEM. *n* = 3

IF analysis of co-expression between p-CaMKII α and NeuN in L4-L6 DRGs revealed that relative to controls, the proportion of p-CaMKII α-positive L4-L6 DRG neurons (stained with NeuN) in DNP rats was markedly increased in week 2 and 3 of STZ-induced diabetes (representative IF images: Figs. 3a, 4a, and 5a, analysis: Figs. 3b, 4b, and 5b).

### Co-expression of P2X3/p-CaMKII α in L4-L6 DRGs

**Fig. 6:**
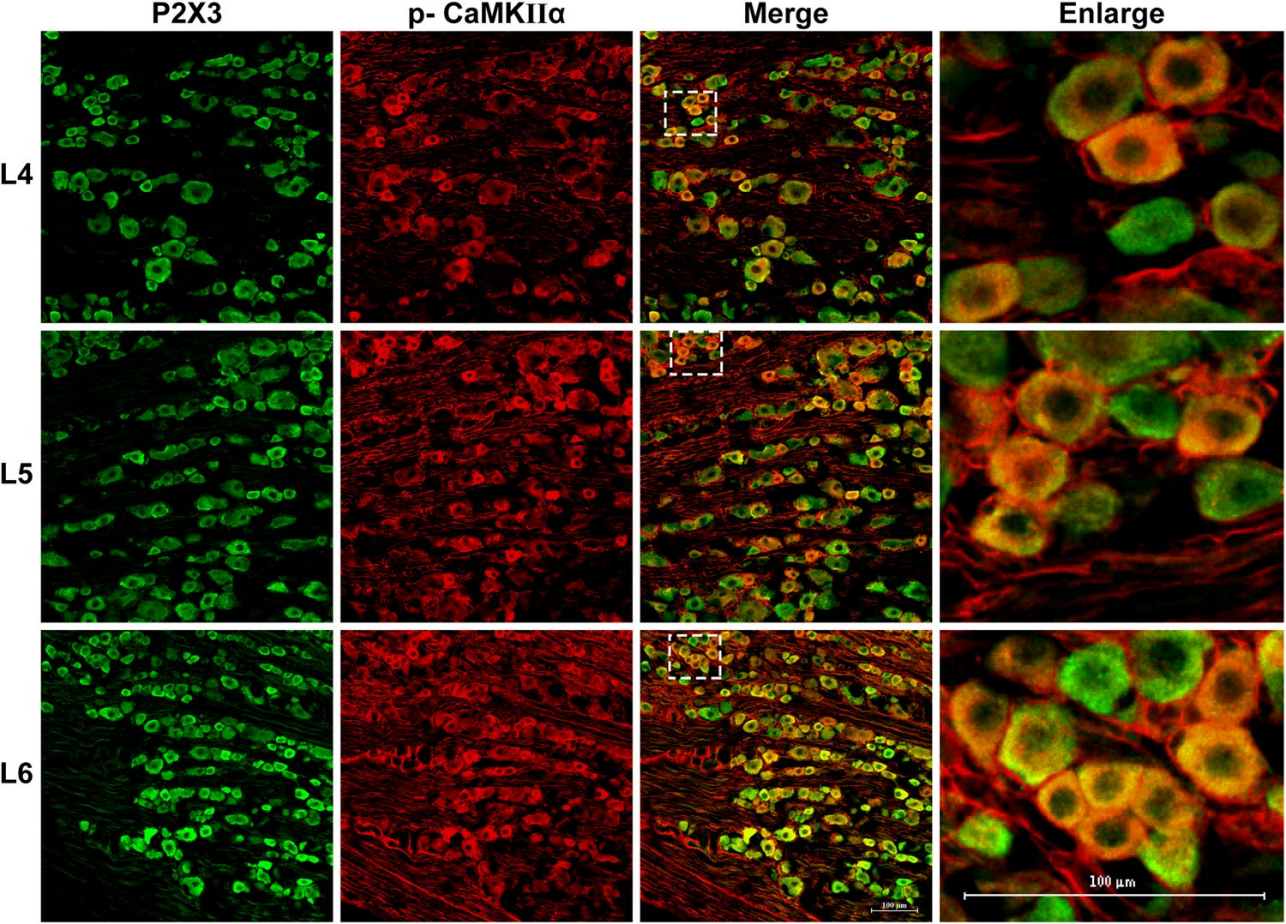
Representative IF images of p-CaMK IIα (red) expression in P2X3 positive (green) in lumbar L4-L6 DRGs of DNP rats. Scale bar 100 µm

IF analysis (Fig. 6) of L4-L6 DRGs from DNP rats revealed P2X3/p-CaMKII α co-expression, implying that p-CaMKII α may interact and modulate P2X3 expression in DRG neurons during DNP.

### Effect of the CaMKII inhibitor KN93 on DNP rats

**Fig. 7:**
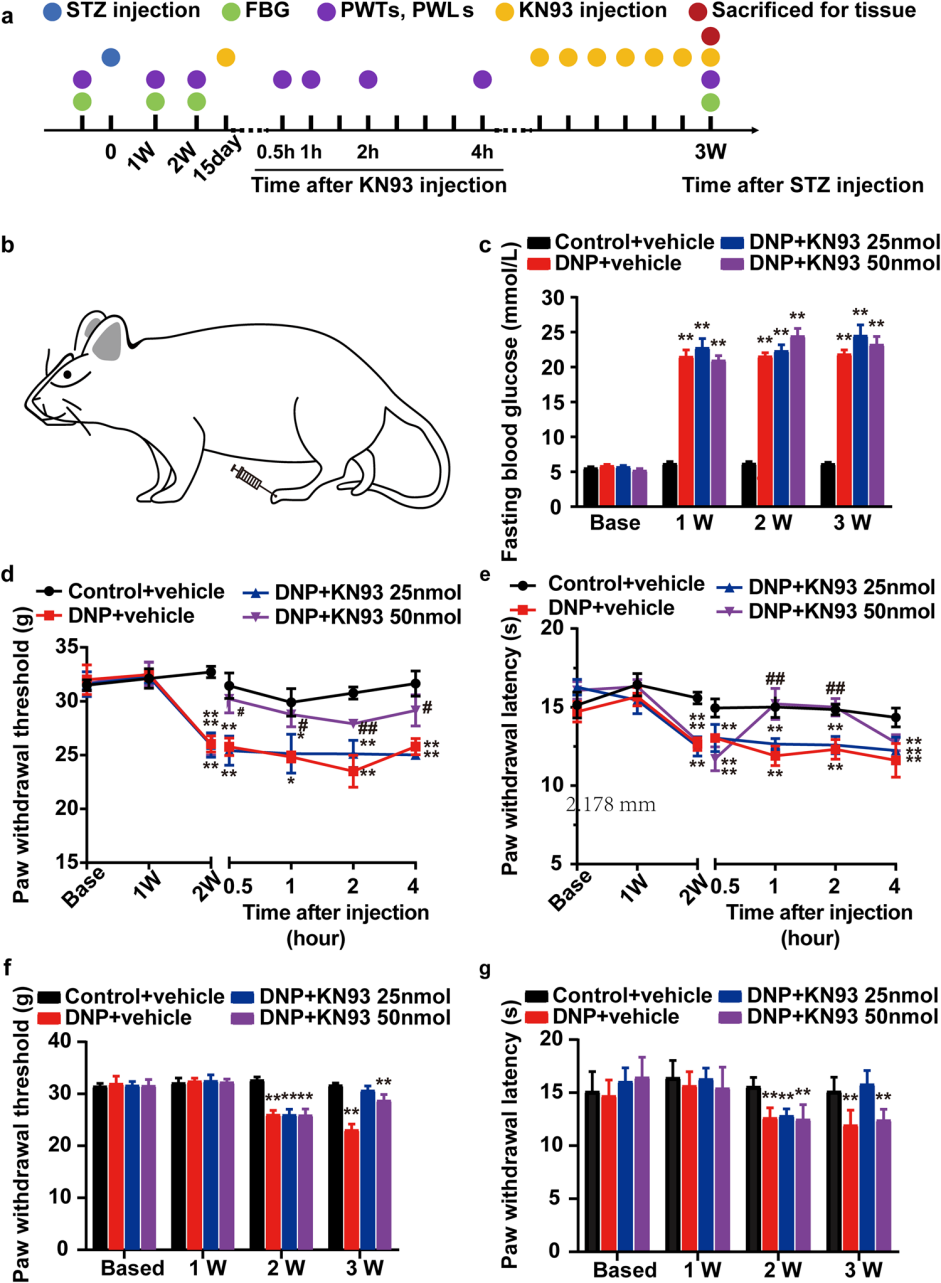
Effects of the CaMKII inhibitor, KN93, on DNP rats. (**a**) Schedule of treatment with KN93. (**b**) A schematic illustration of the injection site. (**c**) Time course effect of STZ injection on FBG. (**d**) Effect of 25 and 50 nmol KN93 on PWT at 0.5, 1, 2, and 4 h after injection. (**e**) Effect of 25 and 50 nmol KN93 on PWL at 0.5, 1, 2 and 4 h after injection. (**f**) Effect of 25 and 50 nmol KN93 on PWT at 3W. (**g**) Effect of 25 and 50 nmol KN93 on PWL at 3W. ^*^P < 0.05, ^**^P < 0.01 vs. Control + vehicle group. ^#^*P* < 0.05, ^##^*P* < 0.01 vs. DNP + vehicle group. *n* = 5

Next, we evaluated the effect of KN93 on DNP rats by examining their PWTs and PWLs. Diabetic rats had significantly higher FBG relative to controls after 1 week, and this difference persisted throughout the experiment (*P* < 0.01, respectively; Fig. 7c), while their PWTs and PWLs decreased significantly decreased on 2 W (Fig. 7d–g), indicating successful establishment of the DNP model. To assess if CaMKIIα phosphorylation contributes to DNP, rats were treated with KN93 or vehicle (0.9% saline) via intraplantar injection on the 2nd week after STZ injection and their PWTs and PWLs evaluated 0.5, 1, 2, and 4 h after KN93 injection. This analysis revealed that relative to the control (vehicle), 50 nmol KN93, but not 25 nmol, significantly reduced mechanical allodynia and thermal hyperalgesia 0.5 h after KN93 injection. This effect on PWT and PWL lasted 4 and 2 h after treatment, respectively (Fig. 7d, e). Relative to control treatment (DNP + vehicle), daily KN93 injection for 7 days (25 and 50 nmol) significantly increased PWT and PWL on week 3 (Fig. 7f, g).

### KN93 reduced CaMKIIα phosphorylation and P2X3 overexpression in the DRGs of DNP rats

**Fig. 8:**
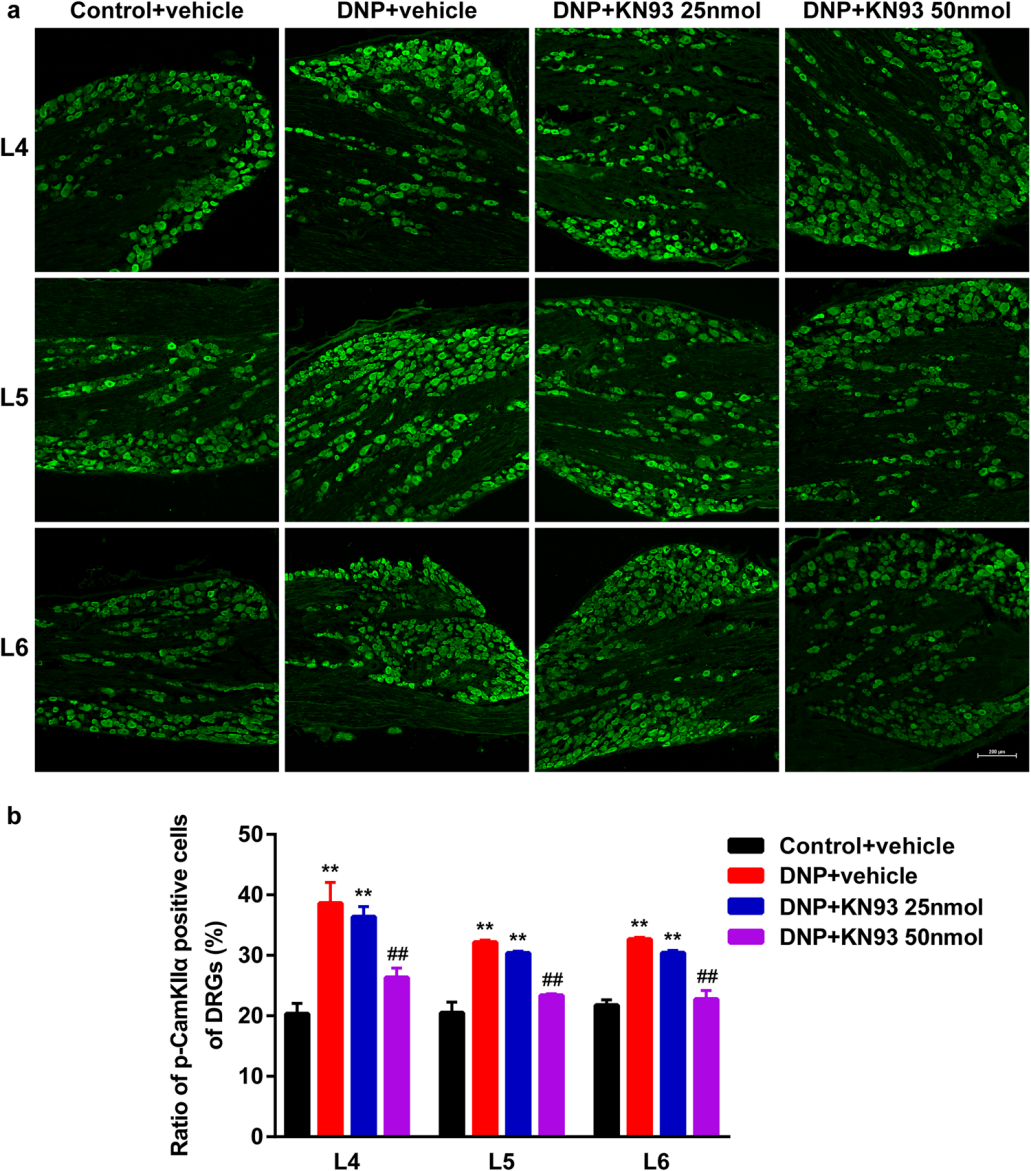
Effect of different KN93 doses on p-CaMKIIα levels in L4-6 DRGs. (**a**) Representative IF images of L4-6 DRGs in Control + vehicle, DNP + vehicle, DNP + KN93 25 nmol, and DNP + KN93 50 nmol groups. Scale bars = 200 μm. (**b**) Proportion of p-CaMKIIα positive cells in L4-L6 DRGs. ^**^P < 0.01 vs. Control + vehicle group. ^##^*P* < 0.01 vs. DNP + vehicle group. Data are presented as mean ± SEM. *n* = 3

**Fig. 9:**
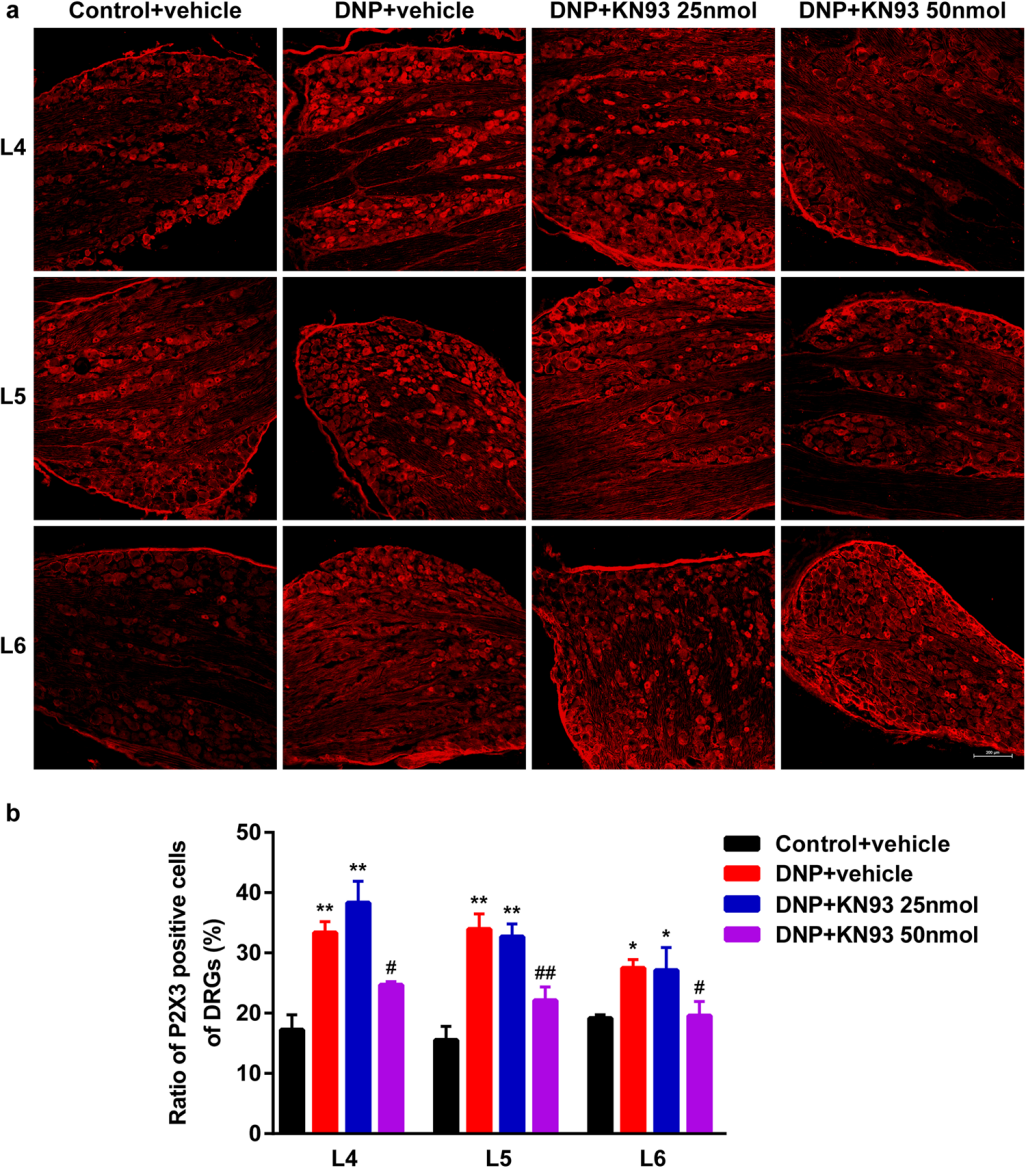
Effect of different KN93 doses on P2X3 levels in L4-6 DRGs. (**a**) Representative IF images of L4-6 DRGs in Control + vehicle, DNP + vehicle, DNP + KN93 25 nmol, and DNP + KN93 50 nmol groups. Scale bars = 200 μm. (**b**) The ratio proportion of P2X3 positive cells in L4-L6 DRGs. ^*^P < 0.05, ^**^P < 0.01 vs. Control + vehicle group. ^#^*P < 0.05,* ^##^*P* < 0.01 vs. DNP + vehicle group. Data are presented as mean ± SEM. *n* = 3

IF analysis of the DRG levels of p-CaMKIIα and P2X3 revealed that they were significantly elevated in the DNP + vehicle group (*P* < 0.01, Figs. 8 and 9). Moreover, treatment with KN93 (50 nmol) significantly reduced p-CaMKIIα and P2X3 levels relative to DNP rats treated with vehicle (*P* < 0.01, Figs. 8 and 9). However, 25 nmol KN93 did not significantly affect p-CaMKIIα and P2X3 levels (*P* > 0.05). These results indicate that CaMKIIα inhibition by KN93 suppressed CaMKIIα phosphorylation and P2X3 upregulation in the DRGs of DNP rats.

## Discussion

There are two types of diabetes based on underlying causes. In general, type 1 diabetes may be directly or indirectly caused by damage to insulin-producing pancreatic β cells due to immune destruction [31, 32]. On the other hand, type 2 diabetes results from insulin resistance [33]. Both types of diabetes are associated with neuropathic pain. STZ is commonly used to induce DNP in experimental animals [34–36] and causes peripheral neuropathy and hyperalgesia when administered at high doses or multiple small doses. Here, we successfully established a rat model of DNP using a single, high dose of STZ. We observed that p-CaMKII α levels were upregulated in the DRG of the DNP rats and that it was co-expressed with P2X3. Treating the DNP rats by intraplantar injection of KN93 [37, 38] at 25 and 50 nmol revealed that the higher dose, but not the lower one, relieved DNP and downregulated p-CaMKIIα and P2X3 levels.

The role of CaMKII in nociceptive processing has been intensively studied. CaMKII is the foundation of synaptic plasticity [39, 40] and plays a key role in pain modulation. CaMKII is expressed in about 50% of rat DRG neurons which regulate pain [20]. Inflammatory pain upregulates CaMKII in DRGs [21] and the spinal cord in a rat model of type 2 DNP [41, 42]. In rodent models of diabetes, increased calcium currents and cytosolic calcium release from internal storage have been observed in peripheral sensory neurons and DRGs [43]. Upon binding to Ca^2+^, CaMKII is activated by auto-phosphorylation. Intrathecal administration of KN93 reduces neuropathic pain [44, 45] and pain-related behavior in DNP rats [41, 42]. Furthermore, silencing CaMKIIα expression effectively relieves both the evoked pain and persistent spontaneous pain [46]. In hyperglycemia, KN93 partially suppresses increased CaMKII phosphorylation [47]. In the present study, western blot analysis revealed elevated p-CaMKIIα levels in L4, L5, and L6 DRGs but CaMKIIα levels did not change significantly. Colocalization IF revealed elevated p-CaMKIIα levels in DRG neurons. Both single and repeated injections of high-dose (50 nmol) KN93, but not low-dose (25 nmol) KN93, significantly relieved DNP and reduced DRG levels of p-CaMKIIα levels. A previous study also showed that KN93 inhibits CaMKII activity dose-dependently [48], which is consistent with our findings that elevated p-CaMKIIα plays a critical role in mediating DNP.

In addition to p-CaMKIIα, P2X3 also contributes to DNP. Our data show that P2X3 expression was increased in the DRG of STZ-induced DNP rats, which is consistent with previous reports [11, 12]. P2X3 is known to mediate neuropathic pain and A-317491, a P2X3 antagonist, suppresses pain signal transmission on primary afferents in the DRGs of a rat model of chronic neuropathic pain [49–51]. A past study found that CaMKII regulates purinergic signaling via intracellular signaling pathway, which in turn modulates P2X3 [25]. Here, based on our previous findings that P2X3 was upregulated in the L4-L6 DRGs of DNP rats [12], we examined the effect of CaMKII on P2X3 in L4-L6 DRGs. Colocalization IF revealed p-CaMKIIα co-expression with P2X3 in DNP rats. High-dose KN93 (50 nmol) reduced p-CaMKIIα and P2X3 levels in DRGs. The latter is widely implicated in neuropathic pain [52–54] and DNP. Thus, p-CaMKIIα may influence DNP by modulating P2X3 expression in DRGs.

## Conclusions

These results revealed that the p-CaMKIIα upregulation in DRG is involved in DNP, which possibly mediated P2X3 upregulation, indicating CaMKIIα may be an effective pharmacological target for DNP management.

## Data Availability

The datasets generated during and/or analyzed during the current study are available from the corresponding author on reasonable request.

## Abbreviations

BW: Body weight
CaMKIIα: Calcium/calmodulin-dependent protein kinase II α
DRG: Dorsal root ganglion
DNP: Diabetic neuropathic pain
FBG: Fasting blood glucose
PWT: Paw withdrawal threshold
PWL: Paw withdrawal latency
STZ: Streptozotocin
